# Case report: ZFYVE19 gene mutation is associated with familial cholestasis

**DOI:** 10.3389/fmed.2024.1400475

**Published:** 2025-02-21

**Authors:** Mei-Yan Xue, Ling-Ling Huang, Yue-Yong Zhu, Qi Zheng

**Affiliations:** ^1^Department of Hepatology, Fujian Clinical Research Center for Hepatopathy and Intestinal Diseases, Hepatology Research Institute, The First Affiliated Hospital, Fujian Medical University, Fuzhou, China; ^2^Department of Hepatology, National Regional Medical Center, Binhai Campus of the First Affiliated Hospital, Fujian Medical University, Fuzhou, China; ^3^Fujian Key Laboratory of Precision Medicine for Cancer, Fujian Key Laboratory of Laboratory Medicine, The First Affiliated Hospital, Fujian Medical University, Fuzhou, China

**Keywords:** cholestasis, ZFYVE19, gene detection, liver cirrhosis, hereditary

## Abstract

The etiology of cholestatic liver disease is complex, with clinical manifestations being nonspecific, and biochemical abnormalities mainly characterized by elevated alkaline phosphatase (ALP) and glutamyl transpeptidase (GGT). Due to the lack of specific symptoms and diverse causes, the diagnosis poses certain challenges. Here, we present a case of liver cirrhosis with predominant cholestatic features of unknown etiology. Despite multiple comprehensive routine etiological screenings and liver biopsies, the diagnosis remained unclear. Subsequent whole exome sequencing revealed the diagnosis of liver cirrhosis caused by familial cholestasis related to a mutation in the ZFYVE19 gene. Through this case report analysis, we aim to broaden the diagnostic approach for cholestatic liver disease of unknown etiology, identify the cause accurately, and intervene promptly.

## Introduction

Cholestatic liver disease is a liver and bile system disorder caused by various reasons that result in the obstruction of bile production, secretion, and excretion, leading to the accumulation of bile in the liver and reflux into the blood, causing a series of organic damage, metabolic disorders, and functional disturbances (1, 2). With the advancement of genetic diagnostic technologies in recent years, the detection rate of genetically-related cholestatic liver diseases has been increasing (3). Previous studies have reported mutations in the ZFYVE19 gene associated with cholestatic liver disease in children (4–6), but there have been few reports in adult patients.

Here, we report a case of adult patient who developed liver cirrhosis due to familial cholestasis caused by ZFYVE19 gene mutation.

## Case report

A 39-year-old male food delivery worker was referred to our hospital in June 2023 due to abnormal liver function for 11 years. During this period, he has repeatedly been found to have elevated transaminase levels during check-ups at other hospitals. Notably, aside from his parents’ consanguineous marriage, his younger brother died from esophagogastric bleeding caused by suspicious Wilson’s disease, but genetic testing was not carried out. The patient had no history of alcohol abuse or metabolic syndrome, and had not taken any other medications, herbal products, or dietary supplements.

**Figure 1:**
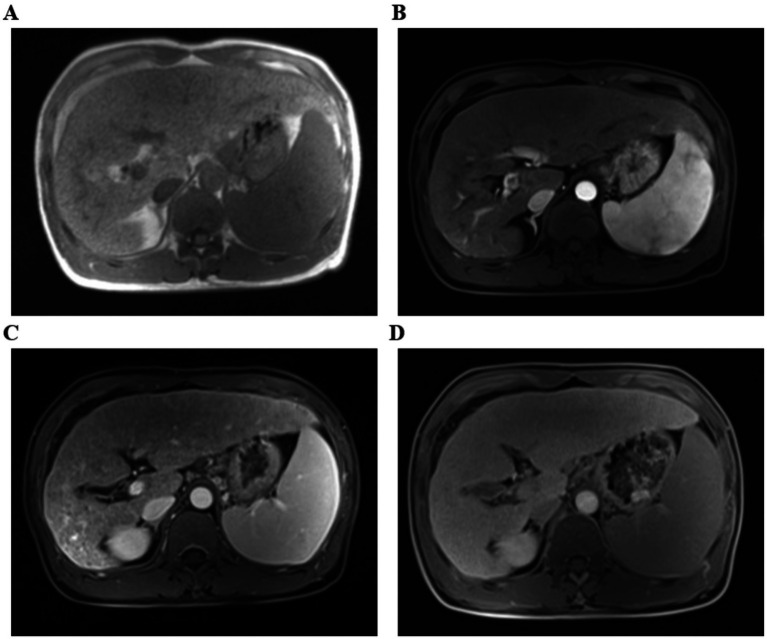
The upper abdominal magnetic resonance imaging (MRI) with T1-weighted sequences. **(A)** Flat phase. **(B)** Arterial phase. **(C)** Portal venous phase. **(D)** Delayed phase. (The arrow indicates that it suggest liver cirrhosis).

The physical examination of the patient was normal. However, multiple liver function tests before admission showed a significant increase in gamma glutamyl transferase (GGT) levels, and mild increases in alanine aminotransferase (ALT), aspartate aminotransferase (AST), alkaline phosphatase (ALP) levels, and total bile acids (see Table 1). Further laboratory tests for hepatitis B surface antigen, hepatitis C virus antibody, hepatitis E virus antibody, antinuclear antibody, antibody spectrum, and various autoantibodies (such as anti-smooth muscle autoantibody, antinuclear autoantibody, antimitochondrial autoantibody, and anti-liver kidney microsomal autoantibody) were negative. Because his younger brother died of Wilson’s disease, a serum ceruloplasmin test was performed, which showed a mild decrease. The copper-expelling test did not show abnormalities, and the ATP7B gene test had no pathogenic variations. Additional tests including iron, and ferritin also showed negative results. The upper abdominal magnetic resonance imaging (MRI) showed the presence of cirrhosis, a small amount of ascites, and splenomegaly (Figure 1). However, Upper gastrointestinal endoscopy did not reveal any gastroesophageal varices.

**Table 1:** The patient’s liver function test results before this hospital admission.

Variable	Normal range	2022.05.17	2022.06.14	2022.07.22	2023.02.14	2023.02.27	2023.06.77
TBIL (μmol/L)	0–26	30.1	33.1	20.2	17.0	16.1	12.8
DBIL (μmol/L)	0–8	13.4	11.6	10.9	14	9.6	7.0
TBA (μmol/L)	0–10	N	N	35.7	N	30.5	N
ALB (g/L)	40–55	39.4	40	41.8	38.8	38	38.8
ALT (U/L)	9–50	135.2	132.7	88	109	68	71
AST (U/L)	15–40	73.4	91.4	77	91	62	67
GGT (U/L)	10–60	746	701.9	578	537	549	574
ALP (U/L)	45–125	167	147.7	136	145	153	144

TBIL, total bilirubin; DBIL, direct bilirubin; TBA, total bile acids; ALB, albumin; ALT, alanine aminotransferase; AST, aspartate aminotransferase; GGT, glutamyl transpeptidase; ALP, alkaline phosphatase.

**Figure 2:**
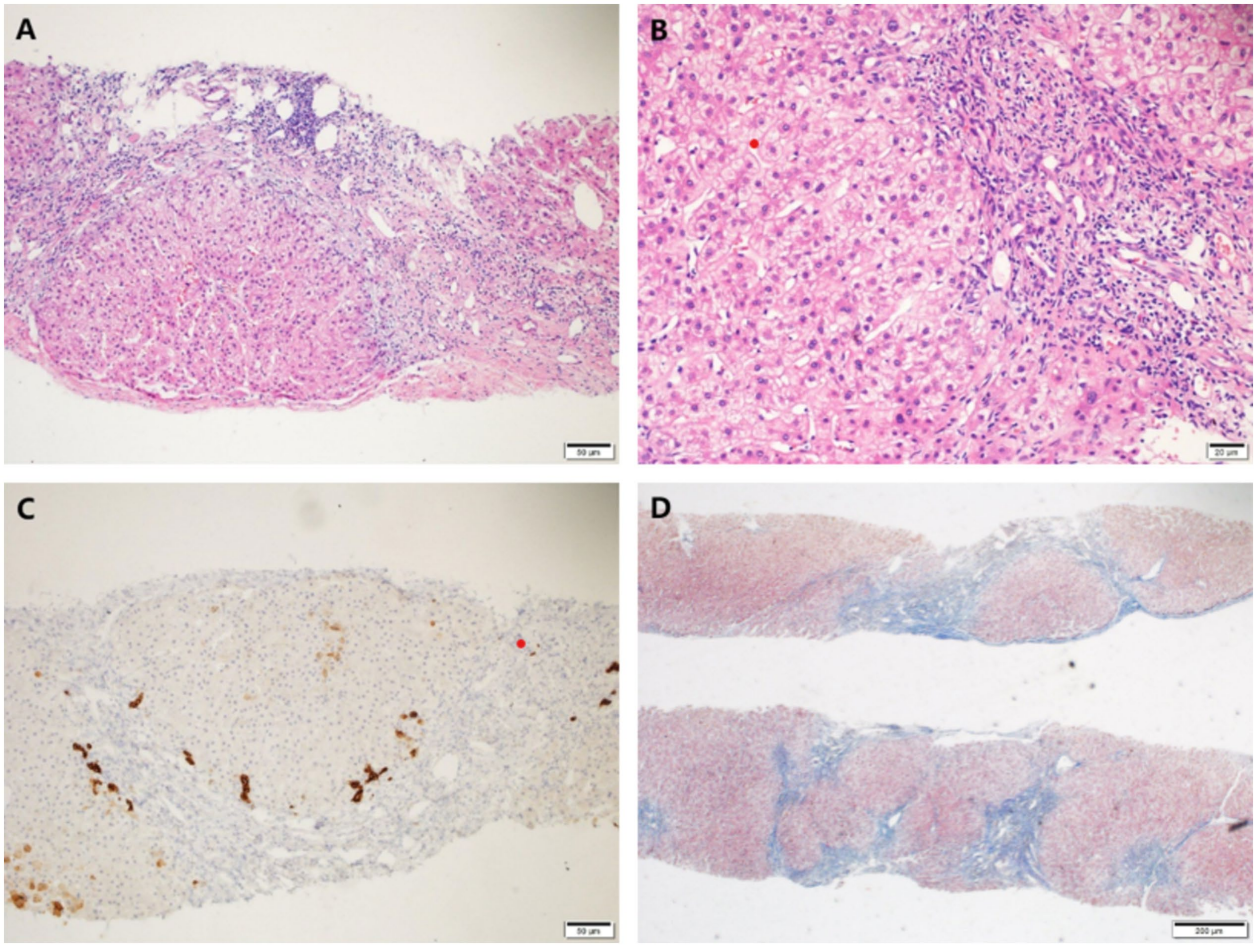
Histological findings of liver biopsy specimens. **(A,B)** H&E staining showed mild interface inflammation and lobular inflammation, as well as enlarged portal areas with fibrous tissue hyperplasia, formation of pseudo-lobules and ductal fibrosis. **(C)** CK7 staining showed partial atrophy of bile duct epithelium without loss. **(D)** Masson staining showed nodular proliferation of liver cells. Original magnification ×100 **(A,C,D)**, ×200 **(B)**.

A percutaneous liver biopsy was also performed. Histological findings of liver biopsy specimens suggested liver cirrhosis with mild lobular inflammation, interfacial inflammation, and hepatocyte ballooning degeneration were encountered (Figure 2).

**Figure 3:**
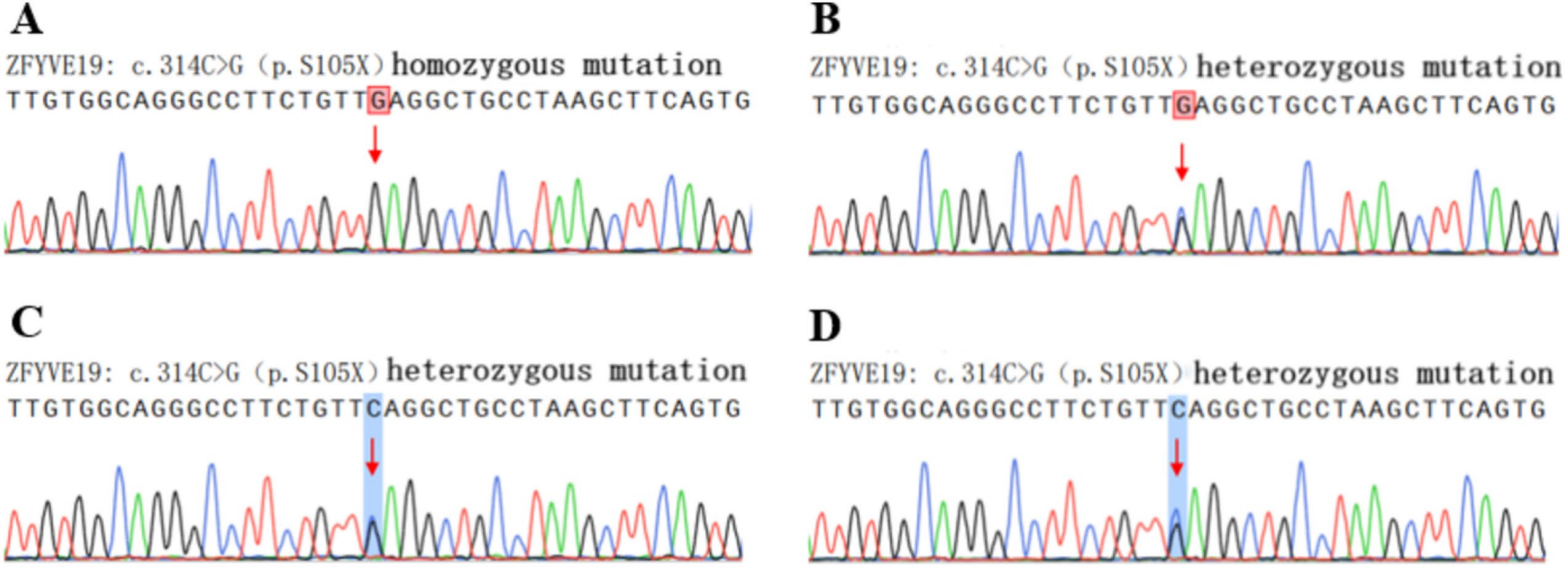
Genetic analysis and familial verification of ZFYVE19. The patient **(A)** carried a homozygous mutation at the c.314C>G site of the ZFYVE19 gene, while his parents **(B,C)** and son **(D)** carried heterozygous mutations.

The cause of liver cirrhosis was still unclear, so genetic testing was performed on both the patient and their family members. Using Sanger technology, the c.314C> site of the ZFYVE19 gene in the patient was verified, revealing a homozygous mutation of ZFYVE19: c.314C>G (p.S105X) (Figure 3). The final diagnosis was liver cirrhosis caused by familial cholestatic liver disease related to ZFYVE19 gene mutation.

The patient regularly received ursodeoxycholic acid (UDCA, 13-15 mg/kg/day) treatment, and his liver function gradually improved during the four-month follow-up. Liver function test results on October 30, 2023 showed a decrease in GGT levels to 181 U/L, with ALT, AST, and ALP levels approximately normal.

## Discussion

The zinc finger FYVE-type containing 19 (ZFYVE19) gene is located on chromosome 15 (15q15.1), coding for the protein ANCHR (6, 7). According to related studies (7), the ANCHR protein contains a FYVE-type zinc finger structure and two type 1 MIT-interacting motifs (MIMs), which can prevent cell division by binding to vacuolar protein sorting 4 (VPS4). VPS4 plays a key role in the transition from the G2 phase of DNA synthesis to metaphase in the cell division cycle. The pathogenesis of liver disease due to mutations in ZFYVE19 genes remains unknown. Luan et al. (6) and Mandato et al. (4) believe that VPS4 is involved in the occurrence of cilia (8, 9). Abnormal binding of ZFYVE19 to VPS4 during mutations may lead to abnormal chromosome segregation and DNA damage, resulting in disruptions to ciliary regulatory pathways, dysplasia of primary cilia, and malfunction of posterior bile duct cells. The pathology presents as portal vein dysplasia, fibrosis of portal veins, hyperplasia, and malformation of bile duct cells.

The main targets of liver diseases caused by ZFYVE19 gene mutations are located in the portal vein and cholangiocytes, thus, cholestasis and portal hypertension are the main clinical manifestations, such as jaundice, pruritus, acute upper gastrointestinal bleeding, and ascites (4, 6, 10). Previous case reports have predominantly involved children and rarely adults (3, 4, 11). However, this case occurred in an adult male whose parents were cousins, and his younger brother died of esophagogastric bleeding caused by suspicious Wilson’s disease. The reason for his delayed onset might be the variation of the gene site. Subsequent diagnostic tests, including liver function analysis, abdominal CT/MRI, gastroscopy, and liver tissue pathology, failed to confirm the pathogen. The final diagnosis was made through whole exome sequencing (WES), confirming that the patient had developed cirrhosis due to a ZFYVE19 gene mutation-related hereditary cholestatic liver disease.

For the treatment of this disease, ursodeoxycholic acid (UDCA, 15–20 mg/kg/day) can improve cholestasis and thus improve liver function (1, 2, 9). Despite UDCA response in patients, some of them still have undergone liver transplantation (LT) for recurrent upper gastrointestinal bleeding, and no obvious complications have been observed in follow-up. Therefore, liver transplantation is considered an effective therapy (4, 6, 12). Furthermore, odevixibat, an inhibitor of the ileal bile acid transporter, has been proven to be effective in treating pruritus (5). In the case described here, the patient is regularly receiving treatment with UDCA, resulting in a significant decrease in GGT and ALP levels compared to baseline. Luan W et al. have demonstrated that ZFYVE19 can greatly improve liver fibrosis and abnormalities in the biliary system at the optimal expression level using a Zfyve19^−/−^ mouse model with adeno-associated virus vector. Gene therapy is expected to be an effective treatment for this disease (13).

In conclusion, we report a special case of cholestasis-ZFYVE19 gene mutation, which broadened the spectrum of genetically related liver diseases. This also confirms that the use of WES may enhance the diagnostic accuracy in cases of undiagnosed cholestasis.

## Data Availability

The datasets presented in this article are not readily available due to patient privacy concerns. Requests to access the datasets should be directed to the corresponding authors.
